# Surveying helix 12 dynamics within constitutively active estrogen receptors using bipartite tetracysteine display

**DOI:** 10.1016/j.jbc.2025.108231

**Published:** 2025-01-27

**Authors:** Lasantha R. Sendanayake, Ranju Pokhrel, Justin M. Holub

**Affiliations:** 1 Department of Chemistry and Biochemistry, Ohio University, Athens, Ohio, USA; 2 Molecular and Cellular Biology Program, Ohio University, Athens, Ohio, USA; 3 Edison Biotechnology Institute, Ohio University, Athens, Ohio, USA

**Keywords:** type 1 nuclear receptor, estrogen receptor alpha, 17β-estradiol, bipartite tetracysteine display, biarsenical profluorophore, protein dynamics, helix 12 transitions

## Abstract

Somatic Y537S and D538G mutations within the estrogen receptor alpha ligand-binding domain (ERα-LBD) have been linked to enhanced cell proliferation, survival, and metastases in ER-positive breast cancers. Such mutations are thought to confer ligand-independent receptor activation by increasing the flexibility of helix 12 (H12), a segment within the ERα-LBD that acts as a dynamic regulator of ERα activity. We employed bipartite tetracysteine display coupled with the biarsenical profluorophore FlAsH-EDT_2_ to monitor ligand-independent structural transitions of H12 in ERα-LBDs that include Y537S or D538G mutations. Our results show that in the absence of 17β-estradiol, Y537S and D538G mutations cause H12 to fold into a “stable agonist” conformation that is similar to liganded (17β-estradiol-bound) wildtype ERα-LBDs. We also observed that stable agonist conformations adopted by unliganded Y537S or D538G mutants resist H12 transitions to inactive states. Taken together, these results indicate that Y537S and D538G mutations endow constitutive activity to the ERα by directly influencing H12 dynamics. Furthermore, our findings provide insight into how Y537S and D538G mutations impart resistance to endocrine or antiestrogen therapies in ER-positive breast cancers.

The estrogen receptor alpha (ERα) is a type I nuclear receptor that regulates cell growth, metabolism, signal transduction, and proliferation. The ERα is activated through the binding of endogenous estrogens, including 17β-estradiol (E2), and exerts its biological effects by controlling expression of estrogen-responsive genes (1). The ERα is organized into five domains: an N-terminal transactivation domain, a DNA-binding domain, a hinge region, a ligand-binding domain (LBD), and a C-terminal domain. Structurally, the ERα-LBD (residues 303–552) is composed of 12 α-helices (H1 to H12) and a small β-sheet, which are arranged in a “sandwich fold” that is well conserved among type I nuclear receptors (2, 3). Structural dynamics studies have indicated that helices H1 through H11 resist large-scale organizational changes upon ligand binding (4, 5). In contrast, the C-terminal H12 (residues 538–548) responds to ligand binding by transitioning from an extended to a folded conformation that associates closely with the globular portion of the receptor (Fig. 1*A*). This so-called “stable agonist” conformation enhances ERα activity by facilitating its dimerization, association with DNA, and recruitment of transcriptional coactivators (1, 5, 6).

**Figure 1:**
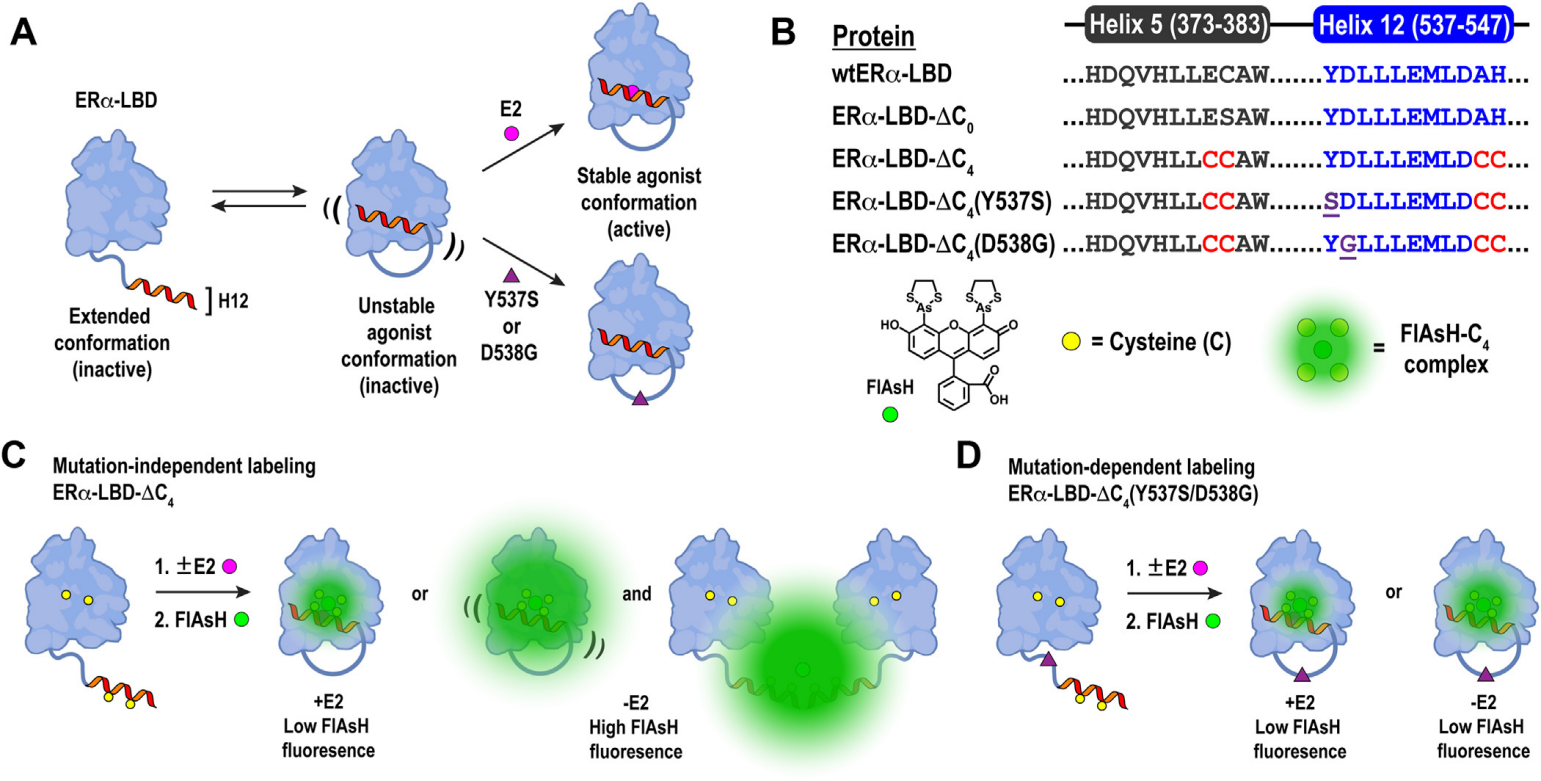
**Monitoring ligand-mediated H12 transitions within the ERα-LBD using bipartite tetracysteine display.***A*, cartoon representing the current model for ERα activation in the presence of estrogenic ligand (E2) or somatic mutations (Y537S or D538G). *B*, identities and sequence alignments of C4-containing ERα-LBDs used in this work; the chemical structure of FlAsH and its association with a C4 motif is shown under the protein sequences. *C*, cartoon representation of mutation-independent labeling of ERα-LBD-ΔC_4_. *D*, cartoon representation of mutation-dependent labeling of ERα-LBD-ΔC_4_(Y537S/D538G). Enhanced FlAsH labeling of ERα-LBD-ΔC_4_ occurs only in the absence of E2, whereas enhanced FlAsH labeling is not observed in ERα-LBD-ΔC_4_(Y537S/D538G), even in the absence of E2. ERα-LBD, estrogen receptor alpha ligand-binding domain.

Evidence has shown that constitutively active ERα leads to enhanced proliferation, survival, and metastases in ER-positive breast cancers (7, 8). Therapeutic regimens for such cancers include selective estrogen receptor modulators or aromatase inhibitors (AIs); however, a significant percentage of ER-positive cancers become resistant upon repeat dosing of selective estrogen receptor modulator- or AI-based therapies (9). This acquired resistance has been linked to somatic mutations in the short loop region between H11 and H12 that replace tyrosine with serine (Y537S) or aspartic acid with glycine (D538G) (8). Indeed, it was found that nearly 25% of patients who received AI treatment for 5 years presented conserved Y537S, D538G, or Y537S/D538G mutations in the gene coding for ERα that were not identified in primary tumors (10, 11, 12). Y537S and D538G mutations are thought to stabilize ligand-independent receptor conformations that have enhanced transcriptional activity (Fig. 1*A*). Furthermore, ligand-independent activation of the ERα can confer resistance to therapies that block the actions of endogenous estrogens (13, 14). Understanding the mechanisms that drive ligand-independent activation of the ERα-LBD will facilitate the development of therapeutics that inhibit deleterious actions of constitutively active ERα.

Our laboratory recently developed a fluorescence-based assay (15) (termed FlAsH-ER) that utilizes bipartite tetracysteine (C4) display (16) coupled with the biarsenical profluorophore FlAsH-EDT_2_ (17, 18) to monitor ligand-mediated H12 transitions within the ERα-LBD. FlAsH-ER employs specialized C4-containing ERα-LBDs (Fig. 1*B*) that bind FlAsH only when H12 is loosely associated with the receptor or in an extended conformation (Fig. 1*C*) (15). In the current study, we applied FlAsH-ER to investigate ligand-independent H12 transitions within ERα-LBDs that contain Y537S and D538G mutations. Our findings indicate that unliganded Y537S and D538G mutants adopt stable agonist conformations that are similar to those observed for liganded wild-type receptors (Fig. 1*D*). These observations also support the hypothesis that Y537S and D538G mutations confer constitutive activity to ERα by altering H12 dynamics. Finally, this work demonstrates that FlAsH-ER can be used to identify mutations within the ERα-LBD that impart therapeutic resistance to ER-positive breast cancers.

## Results

### Rational design of constitutively active ER**α**-LBDs that include C4 tags

We used site-directed mutagenesis to develop two C4-containing ERα-LBDs that include somatic mutations Y537S and D538G. These constructs were named ERα-LBD-ΔC_4_(Y537S) and ERα-LBD-ΔC_4_(D538G), respectively (Figs. 1*B*, S1 and S2, Tables S1 and S2). We also generated two previously reported ERα-LBDs (15), ERα-LBD-ΔC_4_ and ERα-LBD-ΔC_0_, as respective positive and negative controls for the FlAsH-ER assay. In order to mitigate nonspecific labeling of solvent-exposed cysteine thiols (19, 20), native residues C417 and C530 were respectively mutated to Ser and Ala in all ERα-LBDs reported herein (Table S2). Importantly, the spacing of the C4 motifs within the stable agonist conformations of each ERα-LBD was found to be similar to that of the optimized CCPGCC peptide sequence bound to ReAsH (21) (Fig. S3). Furthermore, the stable agonist conformations of H12 adopted by the mutants and wildtype ERα-LBD were found to be nearly identical (Fig. S4). All ERα-LBD mutants adopted structural folds similar to wildtype ERα-LBDs (22) and bound E2 with similar IC_50_ values observed for wild-type receptors (23) (Table 1, Figs. S5 and S6). Taken together, these results indicate that the mutants generated for this study are inherently active and compatible with the FlAsH-ER assay.

**Table 1:** Biophysical data collected for ERα-LBD mutants used in this work

Mutant	E2	*T*_m_ (°C)	IC_50_ (nM)	*k*_on_ (M^−1^ min^−1^)	*K*_app_ (μM)
ERα-LBD-ΔC_0_	−	52.5	ND	ND	ND
ERα-LBD-ΔC_0_	+	ND	46.06 ± 5.40	ND	ND
ERα-LBD-ΔC_4_	−	55.5	ND	26,848 ± 264	0.94 ± 0.56
ERα-LBD-ΔC_4_	+	ND	16.69 ± 4.74	18,591 ± 347	1.22 ± 0.70
ERα-LBD-ΔC_4_(Y537S)	−	51.5	ND	29,433 ± 535	0.90 ± 0.65
ERα-LBD-ΔC_4_(Y537S)	+	ND	15.82 ± 3.13	53,976 ± 921	0.54 ± 0.33
ERα-LBD-ΔC_4_(D538G)	−	52.5	ND	50,266 ± 1178	0.57 ± 0.37
ERα-LBD-ΔC_4_(D538G)	+	ND	19.78 ± 3.71	69,923 ± 716	0.16 ± 0.06

ND, not determined.

### Y537S and D538G mutants adopt stable agonist conformations in the absence of E2

**Figure 2:**
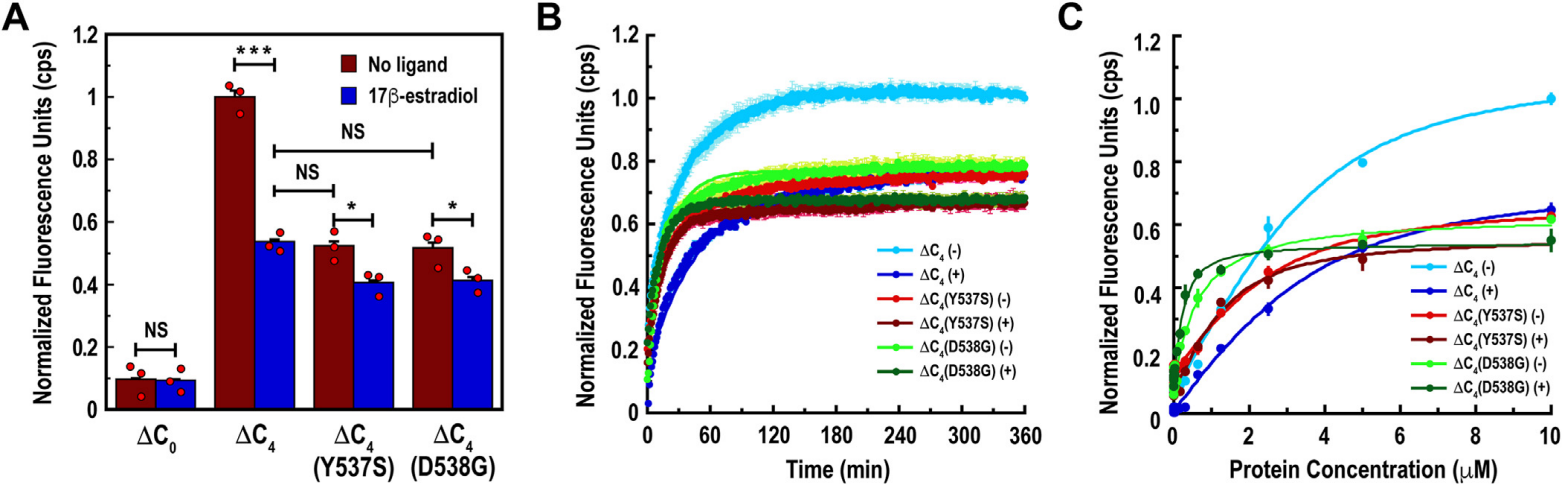
**Results from endpoint-, kinetic- and titration-based FlAsH-ER assays.***A*, endpoint fluorescence of solutions containing FlAsH-EDT_2_ and ERα-LBD mutants in the presence or the absence of E2. Values are displayed as normalized fluorescence units (counts per second, cps). Error bars are standard deviation. ∗*p* < 0.05; ∗∗∗*p* < 0.005; NS, not significant. *B*, initial rates of association and (*C*) equilibrium binding between FlAsH and ERα-LBD mutants in the absence (−) or presence (+) of E2. All data points were collected in triplicate, and each figure represents an average of three independent experiments (*n* = 3). Error bars represent standard deviation. ERα-LBD, estrogen receptor alpha ligand-binding domain.

Once we had developed a set of otherwise functional ERα-LBD mutants, we applied FlAsH-ER to evaluate whether Y537S and D538G mutations affect ligand-independent H12 transitions to stable agonist conformations. For these experiments, we incubated ERα-LBD mutants with FlAsH-EDT_2_ in the presence or absence of E2 and measured the endpoint fluorescence of each reaction (Fig. 2*A*). As expected, ERα-LBD-ΔC_0_ showed minimal fluorescence above background, indicating that FlAsH does not bind ERα-LBDs with no solvent-exposed cysteines. Alternatively, unliganded ERα-LBD-ΔC_4_ exhibited a 10-fold increase in fluorescence compared with ERα-LBD-ΔC_0_. As observed previously (15), we noted a statistically significant decrease in fluorescence for liganded ERα-LBD-ΔC_4_ compared with its unliganded counterpart. We attributed this lower fluorescence intensity to two possible scenarios: (1) inability for H12 to survey conformations that would otherwise be conducive to FlAsH binding (15, 21) and (2) partial occlusion of the C4 motif that forces FlAsH to associate with less than four Cys residues (20, 24). Taken together, these results suggest that H12 in unliganded receptors can survey multiple configurations that are conducive to FlAsH binding, while the liganded complex is locked in a conformation that is less efficient at binding the tracer (Fig. 1*C*).

Upon testing our constitutively active mutants, we determined that unliganded ERα-LBD-ΔC_4_(Y537S) and ERα-LBD-ΔC_4_(D538G) displayed endpoint fluorescence intensities that were nearly identical to that of liganded ERα-LBD-ΔC_4_ (Fig. 2*A*). These results indicate that H12 within unliganded Y537S and D538G mutants adopts a stable agonist conformation that is analogous to liganded wild-type receptors. We also observed that liganded Y537S and D538G displayed fluorescence values that were significantly lower compared with their unliganded counterparts. This suggests that, despite already adopting a stable agonist conformation in the absence of ligand, the binding of E2 to Y537S and D538G mutants further stabilizes the receptor into agonist conformations that are even more restricted (Fig. 1*D*). Indeed, previous studies have shown that the transcriptional activity of constitutively active ERα mutants is further enhanced through E2 binding (10), which is likely because of the ligand becoming trapped in the binding pocket by a highly stable conformation of H12.

To gain further insight into the nature of H12 transitions among our Y537S and D538G mutants, we performed in-gel fluorescence experiments (15, 18) and quantified the percentage of crosslinked dimers within each sample (Fig. S7, Table S3). Our previous work showed that unliganded ERα-LBD-ΔC_4_ can be crosslinked by FlAsH binding (15), which is attributed to a greater percentage of unliganded receptor adopting extended H12 conformations (Fig. 1*C*). As expected, ERα-LBD-ΔC_4_ had a larger percentage (33.2%) of crosslinked dimer in the absence of ligand compared with its liganded counterpart (27.6%). We also found that ERα-LBD-ΔC_4_(Y537S) and ERα-LBD-ΔC_4_(D538G) displayed lower percentages of crosslinked dimer compared with wild-type receptor. Specifically, unliganded Y537S and D538G gave 19.7% and 20.0% crosslinked dimers, respectively, whereas the liganded constructs showed 16.3% and 15.2%. These results further support the notion that Y537S and D538G mutations prevent H12 from adopting extended conformations, even in the absence of ligand.

### FlAsH binds to Y537S and D538G mutants with distinct apparent rate and dissociation constants

To evaluate the kinetics of FlAsH association, we incubated our ERα-LBD mutants with FlAsH-EDT_2_ in the presence or the absence of E2 and measured fluorescence increase as a function of time (Fig. 2*B*, Table 1). In all instances, the time-dependent formation of a FlAsH–receptor complex resulted in an increase in fluorescence that plateaued after 80 min. It was also observed that unliganded receptors displayed higher maximal fluorescence compared with their liganded counterparts upon reaching equilibrium. This observation indicated that FlAsH binds more efficiently to unliganded receptors, regardless of association rate. In addition, unliganded Y537S and D538G mutants gave maximal fluorescence intensity values that were equivalent to those observed with liganded ERα-LBD-ΔC_4_. These results parallel those obtained for our endpoint assay (Fig. 2*A*) and further indicate that unliganded Y537S and D538G mutants adopt stable agonist conformations that are structurally similar to liganded, wildtype ERα-LBDs.

To determine apparent association rate constants (*k*_on_) for FlAsH, all kinetic data were fit to an equation derived from a one-phase association exponential model. The *k*_on_ values for FlAsH binding to ERα-LBD-ΔC_4_ were 26,848 M^−1^ min^−1^ for the unliganded receptor and 18,591 M^−1^ min^−1^ for the liganded complex. This indicates that FlAsH binds ERα-LBD-ΔC_4_ at a slower rate when H12 is locked in a stable agonist conformation. For the Y537S mutant, the *k*_on_ values for FlAsH were 29,433 M^−1^ min^−1^ for the unliganded receptor and 53,976 M^−1^ min^−1^ for the liganded complex. The marginally slower *k*_on_ value for unliganded Y537S indicates that the highly stable agonist conformation adopted by the E2-bound complex was more favorable for binding FlAsH than its unliganded counterpart. Interestingly, the highest *k*_on_ values for FlAsH were observed with the D538G mutant. Here, FlAsH associated with a *k*_on_ value of 50,266 M^−1^ min^−1^ to the unliganded receptor and 69,923 M^−1^ min^−1^ to the liganded complex. The comparatively high *k*_on_ value detected for liganded D538G indicates that the stable agonist conformation adopted by this construct is particularly favorable at binding FlAsH under these conditions.

We next determined apparent equilibrium dissociation constants (*K*_app_) for FlAsH by incubating unliganded and liganded receptors with FlAsH-EDT_2_ and quantifying fluorescence as a function of protein concentration (Fig. 2*C*, Table 1). In all cases, the formation of a FlAsH–receptor complex was accompanied by an observable increase in fluorescence at higher protein concentrations. As was similarly observed for our endpoint and kinetic experiments, unliganded receptors gave higher fluorescence signal compared with their liganded counterparts across all concentrations tested. This trend indicates that FlAsH targets unliganded receptors more efficiently, regardless of binding affinity. It was also noted that unliganded Y537S and D538G mutants gave similar maximal fluorescence intensities compared with liganded ERα-LBD-ΔC_4_. This result further suggests that unliganded Y537S and D538G mutants adopt structural conformations that are akin to liganded wildtype ERα-LBDs.

All *K*_app_ values were determined by fitting the data to an equation derived from first principles with no assumptions. Using this approach, it was determined that unliganded ERα-LBD-ΔC_4_ bound FlAsH with a *K*_app_ of 0.94 μM, whereas the liganded isoform bound FlAsH at 1.22 μM. The comparatively higher binding affinity observed for unliganded ERα-LBD-ΔC_4_ suggests that this construct adopts a structural configuration that is more favorable for binding FlAsH than its liganded counterpart. When testing the Y537S mutant, we found that the unliganded and liganded versions bound FlAsH with respective *K*_app_ values of 0.90 and 0.54 μM. This suggests that the stable agonist conformation adopted by liganded Y537S is more conducive to binding FlAsH than the unliganded isoform. Finally, we found that the D538G mutant had comparatively high affinity for FlAsH, with the unliganded mutant binding at 0.57 μM and the liganded complex binding at 0.16 μM. The higher binding affinity observed with liganded D538G indicates that this isoform adopts a C4 motif that is especially effective at binding FlAsH under these conditions.

## Discussion

In this study, we applied the FlAsH-ER assay (15) to determine how somatic mutations within the ERα-LBD affect ligand-independent structural transitions of H12. Previous work using X-ray crystallography has shown that unliganded Y537S and D538G mutants each fold into stable agonist conformations that align well with the liganded wild-type receptor (8) (Fig. S4). Our findings here provide further empirical evidence that Y537S and D538G mutations facilitate structural transitions to stable agonist states and cause H12 to behave independently of the binding of E2. Importantly, discrete modes of FlAsH binding among the various mutants allowed us to observe subtle variations of H12 dynamics that were not detectable using previously published techniques. For example, we observed that each isoform gave distinct patterns of FlAsH complexation with respect to endpoint fluorescence, association rates, and binding affinities. This suggested to us that each receptor possessed unique C4 geometries that manifested through disparate structural flexibility of H12.

We also observed that higher endpoint fluorescence intensities did not necessarily correlate with faster association rates or stronger binding affinities, which indicates that differential FlAsH-binding modalities can lead to varied fluorescence properties of the tracer (18, 20). For instance, unliganded wildtype ERα-LBD bound FlAsH with moderate association rates and affinity despite having the highest endpoint fluorescence. This indicated that H12 within the unliganded wild-type receptor remains flexible and is relatively slow to bind FlAsH initially. Once bound, however, the unstable agonist or extended conformation of H12 can form C4–FlAsH complexes that fluoresce at relatively high intensity. It was also determined that liganded wildtype ERα-LBD bound FlAsH with the slowest association rate and lowest affinity of any construct tested. This indicated that FlAsH is not able to effectively associate with the stable agonist conformation and was interpreted as H12 within the liganded isoform being comparatively less flexible than the other mutants. Furthermore, the liganded ERα-LBD-ΔC_4_ construct seems to suppress fluorescence once bound, perhaps because of inaccessibility of the full C4 motif (20).

Despite displaying relatively low endpoint fluorescence intensities, FlAsH was found to bind Y537S and D538G mutants more rapidly and with higher affinity compared with wild-type receptors. Once bound to FlAsH, however, the mutant receptors suppressed fluorescence in a manner similarly observed with liganded wildtype ERα-LBDs. This somewhat counterintuitive result suggests that H12 within the mutant receptors remains more flexible and can survey a higher number of configurations that are initially conducive to binding FlAsH, regardless of ligand presence. However, the comparatively lower percentage of FlAsH-crosslinked dimers observed for the Y537S and D538G mutants (Fig. S7, Table S3) indicates that fully extended H12 conformations are less likely and that the enhanced binding of FlAsH results from moderately flexible H12 conformations that are preorganized into stable agonist conformations.

While the inherent advantages of using FlAsH-ER to monitor protein dynamics are apparent, there are some aspects of the technique that may confound its interpretation. For example, the conformational equilibrium of H12 may be shifted by the binding of FlAsH to the C4 motif or the formation of disulfide linkages between C4 thiols. Indeed, the very nature of FlAsH binding necessitates the formation of covalent bonds that may affect the dynamic equilibrium of the labeled protein (25, 26, 27). Nevertheless, our kinetic studies indicated that binding of FlAsH to our ERα-LBDs occurs on the order of minutes, which is in good agreement with previous kinetic studies involving FlAsH binding to other peptides (18). Alternatively, molecular dynamics simulations have shown that H12 transitions within the ERα-LBD occur on the order of nanoseconds (4). The fact that FlAsH association occurs on a much longer timescale indicates that the dynamic equilibrium of the ERα-LBD should be largely unaffected by the biding of FlAsH. In addition, disulfide formation between the C4 thiols is believed to be largely mitigated by the addition of 1 mM Tris(2-carboxyethyl)phosphine (TCEP) to the reaction buffer (28). Moreover, each C4-containing receptor bound E2 with affinities similar to those observed with analogous mutant receptors (8). This indicates that the E2-binding pocket remains accessible in our C4-containing receptors and that H12 is not locked into a stable agonist conformation by superfluous disulfide linkages. Finally, there is the possibility that FlAsH dissociation from the complex may negatively impact the fluorescent readout. Although *k*_off_ rates were not explicitly determined for our FlAsH-ER experiments, previous studies have indicated that the dissociation of FlAsH from C4 motifs takes days to occur (17). The achievement of a stable plateau during our kinetic assays suggests that the reaction has reached equilibrium and that very little FlAsH dissociates from the complex during the timepoints tested. In fact, the binding of FlAsH could only be reversed after the addition of millimolar concentrations of EDT (Fig. S8).

In conclusion, our findings indicate that the stable agonist conformation adopted by Y537S and D538G mutants is ligand independent and reveal a dynamic mechanism for constitutive activation of the ERα. While similar conclusions have been determined previously using techniques such as FRET, hydrogen deuterium exchange mass spectrometry, and molecular dynamics simulations (4, 8), the current study has demonstrated that FlAsH-ER can be applied to determine how somatic mutations affect ligand-mediated H12 transitions within ERα-LBDs. Despite the fact that FlAsH-ER may be perceived as lower resolution than other assays such as FRET or hydrogen deuterium exchange mass spectrometry, FlAsH-ER is rapid, economical, genetically encodable, and can monitor structural transitions within the ERα-LBD with minimal chemical modification to the receptor (8, 29, 30). These attributes endow FlAsH-ER with great potential as a diagnostic screen or, at the very least, as a technique to support higher-resolution assays. We also anticipate that FlAsH-ER will be useful for assessing how various ligands or randomized mutations affect H12 transitions in high throughput. Finally, work in our laboratory is currently focused on adapting FlAsH-ER to monitor H12 transitions in other type I nuclear receptors and applying it in live cells to visualize how ligand-mediated structural changes affect the subcellular trafficking of ERα.

## Experimental procedures

### Endpoint fluorescence

Endpoint fluorescence experiments were performed in triplicate on 384-well black, flat-bottom plates (catalog no.: 3575; Corning) with wells containing 25 μl total reaction volume. The protein (10 μM) was incubated in FlAsH binding buffer (50 mM Tris base, 500 mM KCl, 2 mM DTT, 1 mM EDTA, 1 mM Na_3_VO_4_, 10% glycerol, pH 8.0) supplemented with 1 mM TCEP overnight at room temperature. The binding buffer (1 μl) with or without E2 (final concentration of 10 μM) was then added, and the samples were allowed to incubate at room temperature in the dark for 2 h. Following incubation, 1 μl aliquots of EDT and FlAsH-EDT2 in binding buffer were added at final concentrations of 10 μM and 1 μM, respectively. The reaction mixtures were then allowed to incubate in the dark for 6 h at room temperature. Following incubation, the fluorescence of each sample was measured using a SpectraMax M5e multimode plate reader (Molecular Devices), with an excitation wavelength of 508 nm and an emission wavelength of 530 nm. Data were collected using SoftMax Pro software, version 6.4 (Molecular Devices) and processed using Kaleidagraph, version 4.5 (Synergy).

### Determination of FlAsH apparent rate constants (*k*_on_)

Kinetic experiments were performed on 384-well black, flat-bottom plates (catalog no.: 3575; Corning) with wells containing 25 μl total reaction volume. ERα-LBDs (10 μM) were incubated in 25 μl of binding buffer supplemented with 1 mM TCEP overnight at room temperature. Following incubation, 1 μl of binding buffer with or without E2 (final concentration of 10 μM) was added, and the samples were allowed to incubate at room temperature in the dark for 2 h. Aliquots of EDT and FlAsH-EDT_2_ (1 μl) at respective final concentrations of 10 μM and 1 μM in binding buffer were then added to the samples. The change in fluorescence of each sample was then collected over 6 h with measurements taken at 1 min intervals. Data were collected using a SpectraMax M5e multimode plate reader, with an excitation wavelength of 508 nm and an emission wavelength of 530 nm. The data were processed using Kaleidagraph, version 4.5, and apparent rate constants (*k*_on_) were determined through curve fitting using the equation below:

$$F_{\mathrm{obs}}=F_{\min}+\left(F_{\max}-F_{\min}\right)\left(1-e^{\left(-kT\right)}\right)$$

where *F*_*obs*_ is the observed fluorescence; *F*_*min*_ is the minimum fluorescence value; *F*_*max*_ represents the maximum fluorescence value; *T* is time in minutes; and *k* is the apparent rate constant.

### Determination of FlAsH apparent equilibrium dissociation constants (*K*_app_)

Titration experiments were performed on 384-well black, flat-bottom plates (catalog no.: 3575; Corning) with wells containing 25 μl total reaction volume^.^ ERα-LBDs (10 μM) were incubated in 25 μl of binding buffer supplemented with 1 mM TCEP overnight at room temperature. Binding buffer (1 μl) with or without E2 (final concentration of 10 μM) was then added, and the samples were allowed to incubate at room temperature in the dark for 2 h. The solutions were then transferred to a fresh 384-well black, flat-bottom plate (catalog no.: 3575; Corning) and serially diluted with binding buffer. EDT and FlAsH-EDT2 (1 μl) in binding buffer were then immediately added at final concentrations of 10 μM and 1 μM, respectively. The reactions were then allowed to incubate in the dark for 6 h at room temperature. Fluorescence counts for each sample were then measured using a SpectraMax M5e multimode plate reader, with an excitation wavelength of 508 nm and an emission wavelength of 530 nm. Data were processed using SoftMax Pro software, version 6.4 and Kaleidagraph, version 4.5. The *K*_app_ for each complex was defined by curve fitting using the equation below.

$$F_{obs}=F_{\min}+\left(\frac{F_{\max}-F_{\min}}{2L}\right)(L+P+K_{app}-({\left(L+P+K_{app}\right)}^{2}-{\left(4LP\right))}^{0.5})$$

where *F*_*obs*_ is the observed fluorescence; *P* represents the total protein concentration; *L* is the total concentration of FlAsH-EDT_2_; *F*_*min*_ is the minimum fluorescence value; *F*_*max*_ represents the maximum fluorescence value; and *K*_*app*_ is the apparent equilibrium dissociation constant.

### Statistical analysis

For the data points obtained in each experiment, at least three independent measurements were taken and averaged for the respective receptor in the presence or the absence of ligand. Therefore, each data point represents an average value of three independent experiments (*n* = 3) performed in triplicate; error bars are standard deviations. A two-tailed Student’s *t*-test was used for statistical analysis and a 95% confidence interval was used to denote the significance.

## Data availability

All data described in the article are contained in the main text or the supporting information.

## Supporting information

This article contains supporting information (15, 18, 31, 32, 33, 34, 35).

## Conflict of interest

The authors declare that they have no conflicts of interest with the contents of this article.
