# Supplementary material for: Surveying helix 12 dynamics within constitutively active estrogen receptors using bipartite tetracysteine display
Source: J Biol Chem. 2025 Jan 27;301(3):108231. doi: 10.1016/j.jbc.2025.108231 (PMC11889964; doi:10.1016/j.jbc.2025.108231)
Supplement: Supporting information [file mmc1.docx]

Supporting Information

**Surveying helix 12 dynamics within constitutively active**

**estrogen receptors using bipartite tetracysteine display**

**Lasantha R. Sendanayake^1^, Ranju Pokhrel^1^ and Justin M. Holub^1,2,3,^***

^1^ Department of Chemistry and Biochemistry, ^2^ Molecular and Cellular Biology Program, and ^3^ Edison Biotechnology Institute, Ohio University, Athens, OH 45701, USA.

*Corresponding author: Justin M. Holub, Biochemistry Research Facility 108, 350 W. State St. Athens, OH 45701; E-mail address: [holub@ohio.edu](mailto:holub@ohio.edu)

**Reagents and chemicals.** Tris, bisacrylamide, sodium dodecyl sulfate (SDS), sodium chloride (NaCl), potassium chloride (KCl), lysozyme,1-butanol, and Precision Plus protein marker were purchased from Thermo Fisher Scientific (Waltham, MA). Tetramethyl ethylenediamine (TEMED), ampicillin, β-mercaptoethanol (BME), bovine serum albumin (BSA), sodium orthovanadate (Na_3_VO_4_), polyvinylpyrrolidone (PVP), ammonium persulfate (APS), deoxyribonuclease I (DNAse I), and protease inhibitor cocktail were obtained from Sigma Aldrich (St. Louis, MO). Ethylenediaminetetraacetic acid (EDTA), isopropyl β-D-1-thiogalactopyranoside (IPTG), tris(2-carboxyethyl) phosphine (TCEP), and dithiothreitol (DTT) were purchased from Gold Biotechnologies (St. Louis, MO). LB medium, LB agar, and Coomassie brilliant blue R-250 were obtained from MP Chemicals (Santa Ana, CA). Q5 Hot Start high-fidelity 2x master mix, DpnI, and DH5α competent (high efficiency) *E. coli* cells were purchased from New England Biolabs (Ipswitch, MA). Bacterial protein extraction lysis buffer (PE LB) and yeast PE LB were obtained from G-Biosciences (St. Louis, MO). Centrifugal filtration units and 0.22 μm sterile syringe filters were ordered from Millipore (Darmstadt, Germany). BL21(DE3) cells were purchased from Agilent Life Technologies (Santa Clara, CA). Dimethyl sulfoxide (DMSO) was obtained from Santa Cruz Biotechnology (Dallas, TX). Ni-NTA agarose resin was obtained from Molecular Cloning Laboratories (San Francisco, CA). 17β-estradiol (E2) was obtained from Cayman Chemical Co. (Ann Arbor, MI). Glycerol was obtained from MidSci Chemicals (Fenton, MO). 1,2-ethanedithiol (EDT) was purchased from Fluka (Mexico City, Mexico). Imidazole was obtained from TCI Chemicals (Portland, OR). All primers used for site-directed mutagenesis were purchased from Integrated DNA Technologies (IDT, Coralville, IA). FlAsH-EDT_2_ was synthesized in our laboratory using methods described previously^1,2^. Unless otherwise stated, all other reagents were purchased from commercial sources and used without further purification.

**Site-directed mutagenesis***.* pMCSG7 plasmids coding for ERα-LBD proteins with Y537S and D538G mutations were generated through site-directed mutagenesis on plasmids coding for ERα-LBD-ΔC_4_^2^ (Fig. S1) using Q5 Hot Start high-fidelity 2x master mix (New England Biolabs) according to the manufacturer’s instructions. Briefly, each polymerase chain reaction (PCR) mixture contained 1.25 μL of forward primer (121 ng/μL, Table S1), 1.25 μL of reverse primer (121 ng/μL, Table S1), 4 μL of template DNA (25 ng/μL), 12.5 μL master mix, and 6 μL nuclease-free water. The PCR reaction mixtures were subjected to 35 cycles of PCR amplification using the following conditions: denaturation at 98 °C (30 s), annealing at 67 °C (20 s), and primer extension at 72 °C (4 min ). Once the reaction was complete, 1 μL of DpnI was added to the PCR mixture to digest methylated (parent) DNA and incubated for 1 h at 37 °C. The amplified PCR products were then transformed into the DH5α competent cells (New England Biolabs) following the manufacturer’s protocol. The transformed cells were then spread on agar plates containing 1x ampicillin and incubated at 37 °C overnight. The next day, 100 mL of LB supplemented with 1x ampicillin was inoculated with a single colony picked from the agar plate and incubated at 37 °C overnight with constant shaking at 225 rpm. The cell pellet was then collected from the overnight inoculum and plasmids were purified using a Midi Prep Plasmid Extraction Kit (Qiagen) according to the manufacturer’s guidelines. All purified plasmids were sequenced by the Ohio University Genomics Facility and translated using the web-based Expasy translate tool^3^ (Table S2). Following translation, the plasmids containing positive mutations were stored at -20 °C until further use.


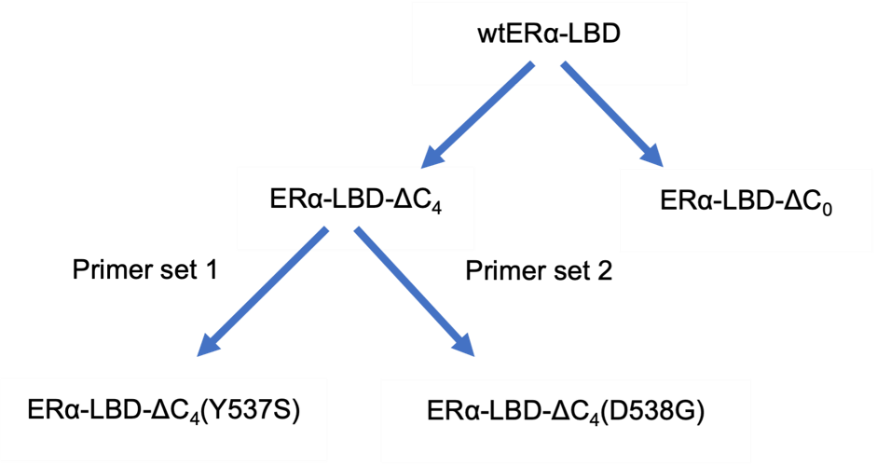


**Figure S1**. Workflow followed to generate ERα-LBD mutants used in this work. The preparation of ERα-LBD-ΔC_4_ and ERα-LBD-ΔC_0_ plasmids from pMCSG7 wtERα-LBD was described previously^2^*.* Herein, plasmids coding for ERα-LBD-ΔC_4_ were subjected to site-directed mutagenesis using primer set 1 and primer set 2 to generate ERα-LBD-ΔC_4_(Y537S) and ERα-LBD-ΔC_4_(D538G), respectively.

**Table S1.** Sequences of primers used for the generation of ERα-LBD mutants.

| **Mutant** | **Primer set** | **Direction** | **Primer sequence** |
| --- | --- | --- | --- |
| ERα-LBD-ΔC_4_(Y537S) | 1 | Forward | 5’-caagaacgtggtgcccctcagtgacctgctgctggagatg-3’ |
|  |  | Reverse | 3’-gttcttggaccacggggagtcactggacgacgacctctac-5’ |
| ERα-LBD-ΔC_4_(D538G) | 2 | Forward | 5’-gtgcccctctatggcctgctgctggag-3’ |
|  |  | Reverse | 3’-ctccagcagcagcaggccatagaggggcac-5’ |

**Table S2.** Sequences of ERα-LBD mutants used in this work; mutated residues are shown in red.

| **Protein** | **Mutations** | **Protein sequence (mutation sites are shown in red)** |
| --- | --- | --- |
| ERα-LBD-ΔC_0_ | C381S; C417S;  C530A | **MHHHHHHSSG VDLGTENLYF QSNAMKRSKK NSLALSLTAD QMVSALLDAE PPILYSEYDP TRPFSEASMM GLLTNLADRE LVHMINWAKR VPGFVDLTLH DQVHLLESAW LEILMIGLVW RSMEHPGKLL FAPNLLLDRN QGKSVEGMVE IFDMLLATSS RFRMMNLQGE EFVCLKSIIL LNSGVYTFLS STLKSLEEKD HIHRVLDKIT DTLIHLMAKA GLTLQQQHQR LAQLLLILSH IRHMSNKGME HLYSMKAKNV VPLYDLLLEM LDAHRLHAPT**  **S** |
| ERα-LBD-ΔC_4_ | E380C; C417S;  C530A; A546C;  H547C | **MHHHHHHSSG VDLGTENLYF QSNAMKRSKK NSLALSLTAD QMVSALLDAE PPILYSEYDP TRPFSEASMM GLLTNLADRE LVHMINWAKR VPGFVDLTLH DQVHLLCCAW LEILMIGLVW RSMEHPGKLL FAPNLLLDRN QGKSVEGMVE IFDMLLATSS RFRMMNLQGE EFVCLKSIIL LNSGVYTFLS STLKSLEEKD HIHRVLDKIT DTLIHLMAKA GLTLQQQHQR LAQLLLILSH IRHMSNKGME HLYSMKAKNV VPLYDLLLEM LDCCRLHAPT**  **S** |
| ERα-LBD-ΔC_4_(Y537S) | E380C; C417S; C530A; Y537S; A546C; H547C | **MHHHHHHSSG VDLGTENLYF QSNAMKRSKK NSLALSLTAD QMVSALLDAE PPILYSEYDP TRPFSEASMM GLLTNLADRE LVHMINWAKR VPGFVDLTLH DQVHLLCCAW LEILMIGLVW RSMEHPGKLL FAPNLLLDRN QGKSVEGMVE IFDMLLATSS RFRMMNLQGE EFVCLKSIIL LNSGVYTFLS STLKSLEEKD HIHRVLDKIT DTLIHLMAKA GLTLQQQHQR LAQLLLILSH IRHMSNKGME HLYSMKAKNV VPLSDLLLEM LDCCRLHAPT**  **S** |
| ERα-LBD-ΔC_4_(D538G) | E380C; C417S; C530A; D538G; A546C; H547C | **MHHHHHHSSG VDLGTENLYF QSNAMKRSKK NSLALSLTAD QMVSALLDAE PPILYSEYDP TRPFSEASMM GLLTNLADRE LVHMINWAKR VPGFVDLTLH DQVHLLCCAW LEILMIGLVW RSMEHPGKLL FAPNLLLDRN QGKSVEGMVE IFDMLLATSS RFRMMNLQGE EFVCLKSIIL LNSGVYTFLS STLKSLEEKD HIHRVLDKIT DTLIHLMAKA GLTLQQQHQR LAQLLLILSH IRHMSNKGME HLYSMKAKNV VPLYGLLLEM LDCCRLHAPT**  **S** |

**Plasmid transformation and glycerol stock preparation.** Plasmids containing positive mutations were transformed into BL21(DE3) cells (Agilent Life Technologies) according to the manufacturer’s guidelines. Following the transformation, the cells were plated on LB agar plates containing 1x ampicillin and grown overnight at 37 °C. The next day, a 5 mL LB medium containing 1x ampicillin was inoculated with a single, isolated colony, and the inoculum was incubated at 37 °C overnight with constant shaking at 225 rpm. For each mutant, glycerol stocks were prepared by adding 500 μL of bacterial transformant inoculum to 500 μL 50% (v/v) glycerol in water. All glycerol stocks were frozen and stored at -80 °C until further use.

**Protein expression and purification.** His-tagged (His_6_) ERα-LBD mutants used in this study were expressed and purified from BL21(DE3) cells using techniques described previously^2,4^. Briefly, transformed BL21(DE3) cells from glycerol stocks were streaked on agar plates containing 1x ampicillin and incubated overnight at 37 °C. A single colony was isolated and used to inoculate a 5 mL LB media starter culture, which was incubated overnight at 37 °C with constant shaking at 225 rpm. Following incubation, the full volume of starter culture was used to inoculate 1 L of LB media containing 1x ampicillin and was incubated at 37 °C with constant shaking at 225 rpm. The culture was grown to an OD_600_ of 0.6 as determined using a UV/Vis spectrophotometer (Cary). Upon reaching the proper OD, the 1 L bacterial culture was cooled to 16 °C with constant shaking at 225 rpm for 20 min. Protein expression was then induced by adding IPTG to the culture at a final concentration of 0.1 mM. Following the addition of IPTG, the culture was incubated at 16 °C with constant shaking at 225 rpm for an additional 18 h. At the end of the incubation, 250 mL fractions were collected and pelleted by centrifugation at 3600 rpm for 20 min at 4°C; the supernatant was then discarded, and all pellets were stored at -80 °C until further use.

Before extracting the expressed protein, all flasks, transfer pipettes, storage tubes, and columns used in the purification process were coated with PVP by soaking them overnight in a 1% (w/v) aqueous PVP solution. Following overnight soaking, the tubes were left to dry completely at ambient temperature (approximately 25 °C). Protein purification was initiated by thawing a bacterial pellet (250 mL) on ice for 30 min. Once the pellet was thawed, it was resuspended in 8.75 mL of lysis buffer (3:1 bacterial PE LB: yeast PE LB (v/v), 50 U DNAse I, 2.5 mg lysozyme, 10 mM imidazole, 2 M urea, 10 mM BME, 25 μL protease inhibitor cocktail, and 10% glycerol, pH 8.0). Following resuspension, the pellet was allowed to incubate with end-over-end rotation at ambient temperature for 20 min on a rotisserie tube rotator. The lysate was then centrifuged twice, first at 40,000 x *g* for 45 min at 4 °C and again at 40,000 x *g* for 30 min at 4 °C. Following the second centrifugation, the cleared supernatant was filtered on ice using a 0.22 μm vacuum filtration unit (Steriflip). Meanwhile, a His_6_ affinity column was prepared by loading 2 mL Ni-NTA resin suspension into a fritted 10 mL PPE column (Thermo) and washing the resin twice with 10 mL loading buffer (3:1 bacterial PE LB: yeast PE LB (v/v), 100 mM imidazole, 2 M urea, 1 mM BME, pH 8.0). The cleared supernatant was then added to the equilibrated column and incubated with end-over-end rotation on a rotisserie tube rotator for 45 min at 4°C. The resin-bound protein was then washed five times with 10 mL wash buffer (50 mM Tris base, 500 mM NaCl, 20 mM imidazole, 15 mM BME, and 10% glycerol, pH 8.0). Following washing, the protein was eluted from the column with 15 mL of elution buffer (50 mM Tris, 500 mM NaCl, 250 mM imidazole, 15 mM BME, and 10% glycerol, pH 8.0) in separate 3 mL fractions. The protein purity was then evaluated by loading samples from each elution onto a 14% polyacrylamide gel and separating them by SDS-PAGE; proteins were visualized by staining the gels with Coomassie blue (Fig. S2). All fractions containing pure protein were combined and dialyzed in 3 L FlAsH binding buffer (50 mM Tris base, 500 mM KCl, 2 mM DTT, 1 mM EDTA, 1 mM Na_3_VO_4,_ and 10% glycerol, pH 8.0) for 48 h at 4 °C with one complete buffer change at 24 h. Following dialysis, proteins were concentrated using centrifugal filtration units (Millipore) to a final concentration of approximately 20 μM, all concentrations were quantified using a Bradford assay^5^. Concentrated proteins were then aliquoted to fresh, pre-chilled microfuge tubes, flash-frozen, and stored at -80 °C until further use.


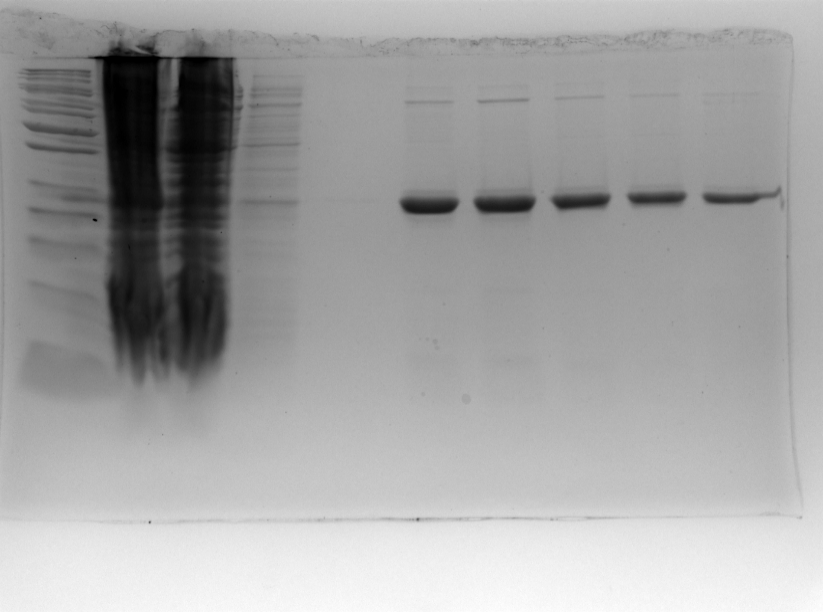


M SL FT W1 W5 E1 E2 E3 E4 E5

kDa

200

85

70

50

40

30

25

20

15

10

**Figure S2**. Representative image showing purified fractions of ERα-LBD-ΔC_4_(D538G) on a 14% polyacrylamide gel following SDS-PAGE. M: marker; SL: sample load; FT: flowthrough; W1 and W5: washes 1 and 5; E1-E5: elutions 1 through 5. Size marker (kDa) is shown to the left of the gel.

**Molecular modeling.** All ERα-LBDs were modeled and aligned using the PyMol Molecular Graphics System v2.1 (Schrodinger). Published structures of ERα-LBDs (PDB IDs: 1ERE, 3UUD, 2B23, 4PXM, and 4Q13) were prepared for analysis by mutating residues E380, A546 and H547 to Cys. Distances between the sulfur atoms were then measured to assess whether the respective C4 motifs occupy spatial arrangements that can be bound by FlAsH (Fig. S3). Structural alignments between H12 domains (residues 537-547) of active ERα-LBDs were also performed to determine whether somatic mutations cause significant structural perturbations of H12 compared to wild-type (Fig. S4).

**
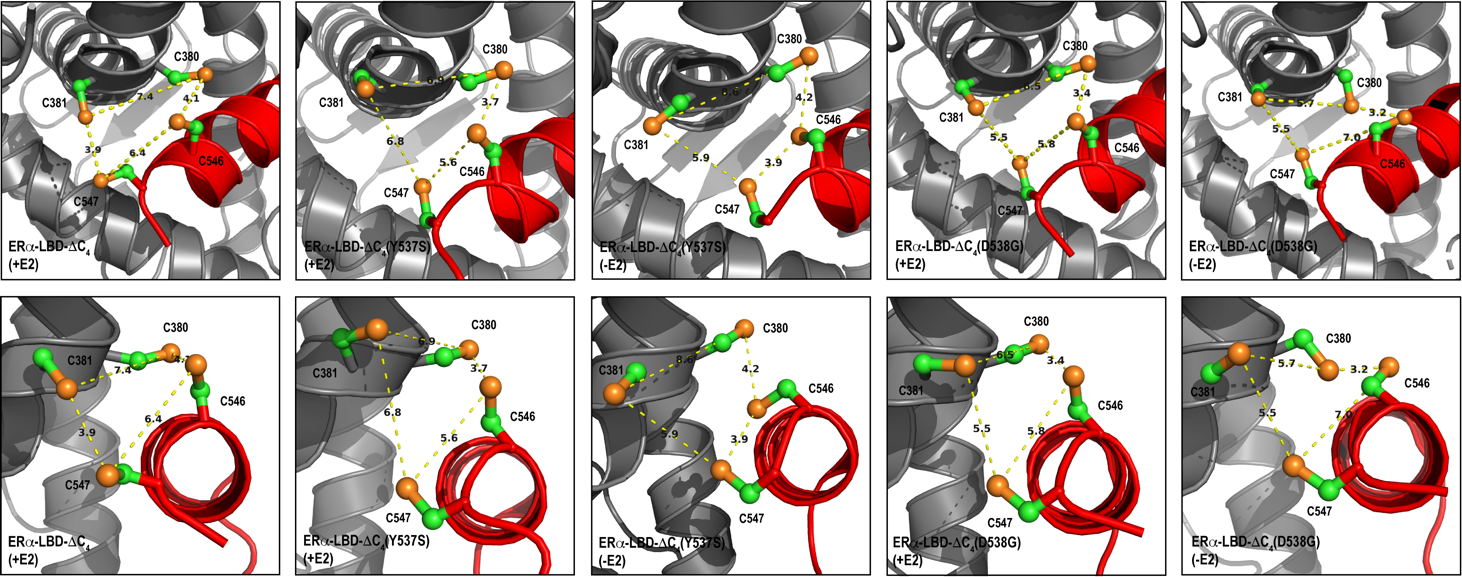
**

**Figure S3**. C4 motifs formed between H12 and the globular portion of the ERα-LBD in the presence (+) and absence (-) of E2. Images (from left to right) are modeled from respective PDB entries: 1ERE, 3UUD, 2B23, 4PXM and 4Q13. H12 is colored red; the ERα-LBD is shown in grey; Cys thiols are rendered as ball and stick; distances (Å) between Cys thiols are indicated by dotted yellow lines.


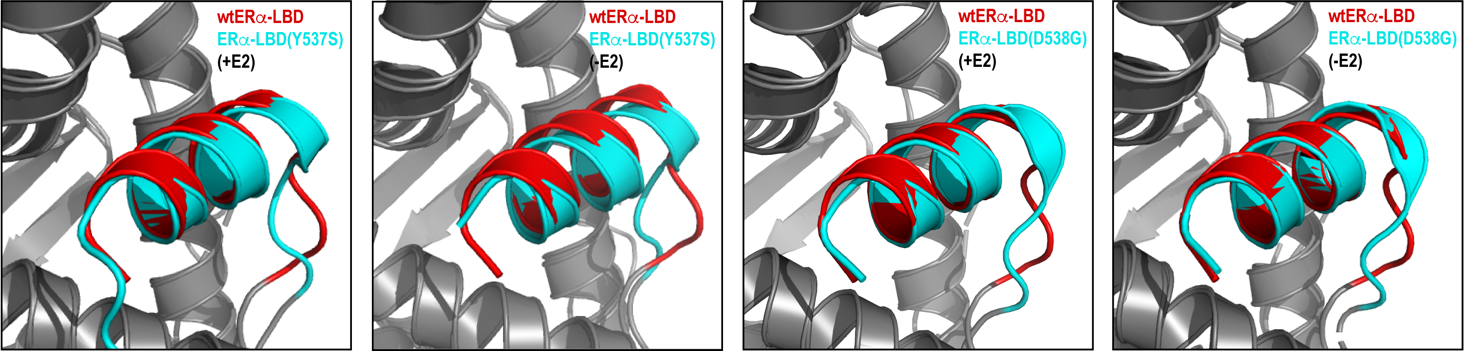


**Figure S4.** Overlayed H12 regions of active wild-type and mutant ERα-LBDs in the presence (+) and absence (-) of E2. Images (left to right) are modeled from PDB entries: 1ERE overlayed with 3UUD, 2B23, 4PXM and 4Q13. Color of H12 and protein identity are shown in the figure legend.

**Circular dichroism spectropolarimetry.** The solution-phase structures of our ERα-LBD mutants were analyzed by wavelength-dependent circular dichroism (CD) spectropolarimetry using a Jasco J-715 CD spectropolarimeter interfaced with a Peltier temperature control unit. For CD analysis, stock protein solutions were diluted to a final concentration of 3.5 μM in CD buffer (20 mM Tris, 100 mM NaCl, pH 8.0). For wavelength scans, the protein solutions were equilibrated for 10 min at 20 °C in the sample chamber before being analyzed. All spectra were generated by collecting the CD (mdeg) of each protein from 250 nm to 190 nm at 20 °C (Fig. S5). Each spectrum represents a background subtracted (buffer only), average of four scans. Temperature-dependent CD scans were obtained from solutions containing 3.5 μM protein diluted in CD buffer (20 mM Tris, 100 mM NaCl, pH 8.0). For thermal scans, solutions were allowed to equilibrate at 5 °C for 10 min in the sample chamber before being analyzed. Thermal melts were generated by monitoring the CD (mdeg) of each protein at 218 nm as the temperature was increased from 5 to 95 °C at 1 °C min^-1^ intervals (Fig. S5, insets). The midpoint of the resultant sigmoidal curve was determined by taking its first derivative, which was used to define the melting temperature (Tm) of each protein. All spectra were generated using J-700 Software v1.5 (Jasco) and processed using Kaleidagraph v4.5 (Synergy).


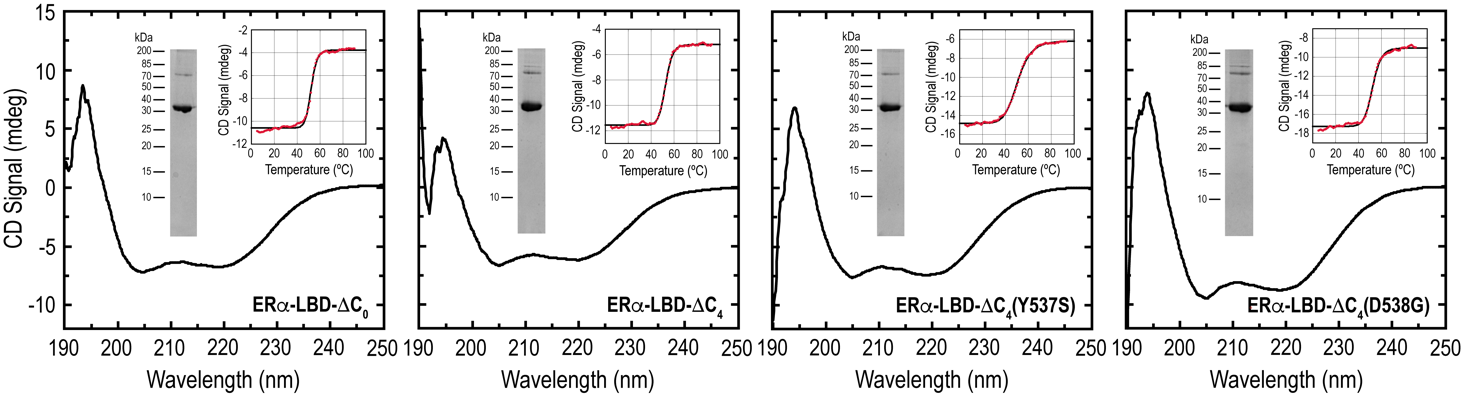


**Figure S5**. Circular dichroism (CD) spectra and SDS-PAGE of recombinant ERα-LBD proteins. All spectra show far-UV CD signatures typical of proteins with α-helical character. SDS-PAGE gels show single bands at the expected molecular weight of the recombinant proteins. Thermal unfolding of proteins (insets) was monitored at 218 nm and plotted as a function of increasing temperature.

**Competitive binding assays.** The binding affinity of E2 to our ERα-LBD mutants was determined using a PolarScreen^TM^ ER Alpha Competitor Assay, Red (Life Technologies) according to the manufacturer guidelines (Fig. S6). Here, ERα-LBD proteins were diluted in ER Red Screening Buffer to a final concentration of 154 nM and pre-incubated with Fluormone^TM^ tracer (3 nM) for 45 min. This mixture was used as a 2x stock ERα-LBD/Fluormone^TM^ complex solution to prepare the final assay plate. In the meantime, E2 was serially diluted in DMSO from 1 mM to 69 pM, each 2 μL of E2 from the dilution series was then added to 98 µL ER Red Screening Buffer to make a concentration series of 20 μM to 1.38 pM. Following incubation, 10 μL of 2x ERα-LBD/Fluormone^TM^ complex was transferred in triplicate into each respective well of a 384-well, black, low volume, round-bottom plate (#3575, Corning). 10 μL aliquots of E2 (serially diluted in ER Red Screening Buffer) were then added to the wells containing the pre-bound ERα-LBD/Fluormone^TM^ complex. The reaction mixture was then allowed to incubate for 2 h at ambient temperature. Following incubation, the fluorescence polarization (mP) of each solution was measured using a Cytation 3 plate reader (Bio Tek Instruments, Inc.) outfitted with a red FP filter cube (excitation/bandwidth: 530/25 nm, emission/bandwidth: 590/35 nm). Data were collected using Gen5 software v2.09 (Bio Tek) and processed using Kaleidagraph v4.5 (Synergy Software). The IC_50_ value for each mutant was determined using the equation below:

$$F_{obs}=\frac{{FP}_{max}{-F}_{min}}{\left[ 1+\left( \frac{{IC}_{50}}{L} \right)^{m} \right]}+ F_{min}$$

Where *F_obs_* is the observed polarization value; *F_max_* is the maximum polarization value*; F_min_* is the minimum polarization value; *L* is the concentration of ligand and the *IC_50_* is the half maximal inhibitory concentration. The steepness of the curve is described by the slope factor, *m*.

~~
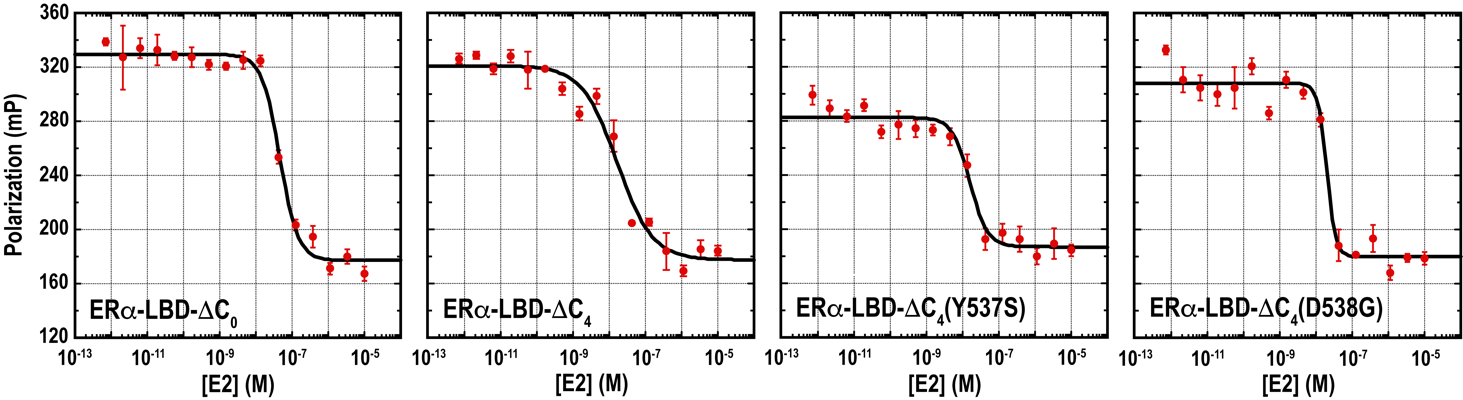
~~

**Figure S6**. Competitive binding assays for ERα-LBD mutants used in this work. Results show fluorescence polarization in millipolarization units (mP) plotted against the concentration of unlabeled ligand (E2). Data are shown for each concentration as a mean of three independent experiments; error bars represent standard deviation.

**In-gel fluorescence of FlAsH-protein complexes.** The ability for FlAsH to label and crosslink ERα-LBD-ΔC_4_ proteins was assessed using in-gel fluorescence as described previously^2,6^ . Briefly, 25 μL volumes of 10 μM ERα-LBD-ΔC_0_, ERα-LBD-ΔC_4_, ERα-LBD-ΔC_4_(Y537S), or ERα-LBD-ΔC_4_(D538G) proteins were pre-incubated in FlAsH binding buffer supplemented with 1 mM TCEP overnight at ambient temperature in individual wells of a 384-well black, flat-bottom plate (#3575, Corning). Following overnight incubation, E2 in binding buffer (10 μM) or binding buffer only (no ligand) was added, and the mixtures were allowed to incubate at ambient temperature for 2 h. Prior to addition, E2 was dissolved in 100% DMSO and further diluted in 20% DMSO (v/v) in FlAsH binding buffer. Following complexation, freshly-prepared solutions of EDT and FlAsH-EDT_2_ in FlAsH binding buffer were added to the wells at final respective concentrations of 10 μM and 5 μM. The reactions were then allowed to incubate in the dark for 6 h at ambient temperature. Following incubation, the samples were mixed with 5× SDS sample buffer (0.312 M Tris, 10% (w/v) SDS, 20 mM TCEP, 0.05 % (w/v) bromophenol blue, and 40% (v/v) glycerol, pH 6.8) and loaded onto a 14% polyacrylamide gel for separation by SDS-PAGE. The separated proteins were then visualized under UV illumination on a Bio-Rad ECL/fluorescence gel imaging cabinet (Hercules, CA). Images were captured using the Pro-Q Emerald 300 protocol provided with Bio-Rad Image Lab software version 5.2.1 (Fig. S7). Staining of the gel with Coomassie blue confirmed the presence of ERα-LBD proteins (data not shown). Quantification of band intensity was performed using ImageJ software^7^ and the results are outlined in Table S3.

**
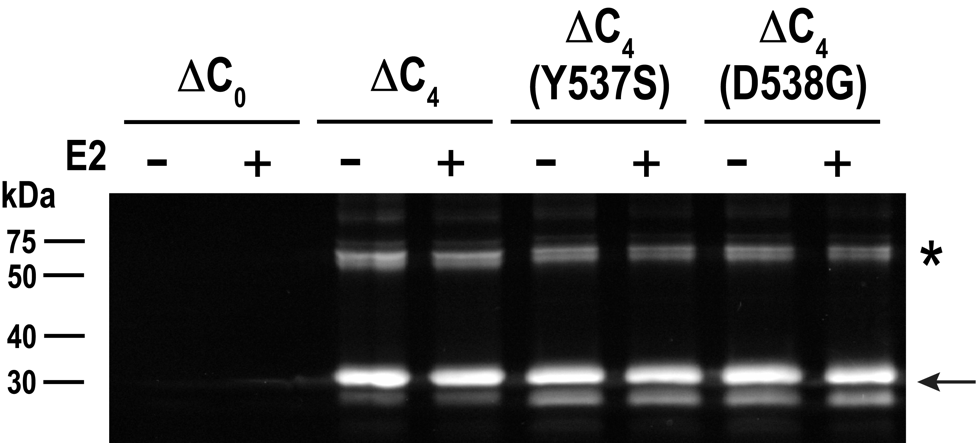
**

**Figure S7.** In-gel fluorescence of various ERα-LBD proteins complexed with FlAsH in the presence (+) or absence (-) of E2. Fully-reduced ERα-LBD proteins were incubated with or without ligand and complexed with FlAsH before being separated by SDS-PAGE. Protein-FlAsH complexes were then visualized by in-gel fluorescence. Monomeric LBDs are indicated with an arrow and the position of presumed crosslinked (dimeric) receptors are indicated with an asterisk (*). Size marker (kDa) is to the left of the gel. Treatments are indicated above each lane; E2: 17β-estradiol.

**Table S3.** Percentage of ERα-LBD species determined by in-gel fluorescence.

| **Protein** | **E2** | **% Dimer** | **% Monomer** |
| --- | --- | --- | --- |
| ERα-LBD-ΔC_4_ | - | 33.17 | 66.83 |
| ERα-LBD-ΔC_4_ | + | 27.56 | 72.44 |
| ERα-LBD-ΔC_4_(Y537S) | - | 19.74 | 80.26 |
| ERα-LBD-ΔC_4_(Y537S) | + | 16.30 | 83.70 |
| ERα-LBD-ΔC_4_(D538G) | - | 20.00 | 80.00 |
| ERα-LBD-ΔC_4_(D538G) | + | 15.18 | 84.82 |

**Reversibility of FlAsH binding.** The reversibility of FlAsH binding to ERα-LBD-ΔC_4_ was tested using kinetic-based FlAsH-ER assays (Fig. S8). For these studies, experiments were performed in triplicate on 384-well black, flat-bottom plates (#3575, Corning), with individual wells containing 25 μL total reaction volume. ERα-LBDs (10 μM) were initially incubated in 22 μL of FlAsH binding buffer supplemented with 1 mM TCEP overnight at room temperature. Following overnight incubation, 1 μL of FlAsH binding buffer alone or FlAsH binding buffer containing E2 at final concentration of 10 μM was added to the wells, and the samples were allowed to incubate at ambient temperature in the dark for an additional 2 h. Following incubation, 1 μL aliquots of EDT and FlAsH-EDT_2_ at respective final concentrations of 10 μM and 1 μM in FlAsH binding buffer were added to the wells. The change in fluorescence intensity of each sample was collected over 6 h with measurements taken at 1 min intervals. At the 6 h timepoint, a 1 μL aliquot of EDT in FlAsH binding buffer was added to each sample at a final concentration of 5 mM. Fluorescent data was then collected for an additional 6 h. All data were generated using a SpectraMax M5e multi-mode plate reader (Molecular Devices), with an excitation wavelength of 508 nm and an emission wavelength of 530 nm. The data were processed using Kaleidagraph v4.5 (Synergy).

**
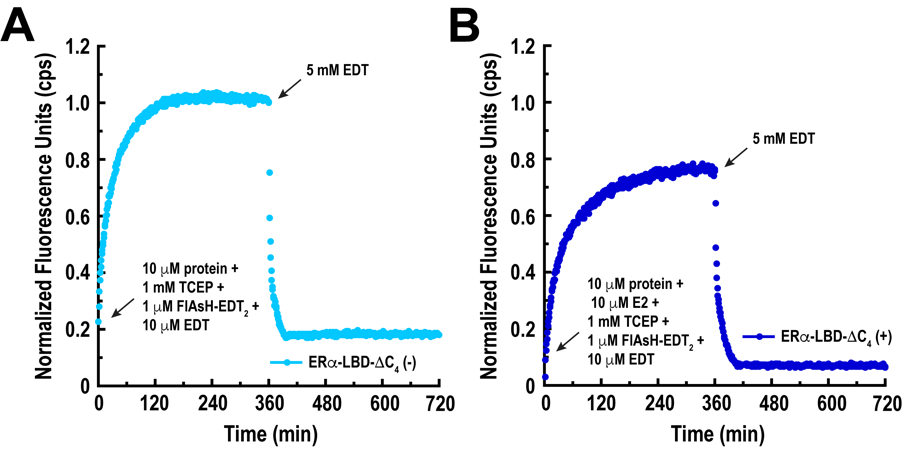
**

**Figure S8**. Kinetics of FlAsH binding to ERα-LBD-ΔC_4_ in the absence (A) or presence (B) of 10 μM E2. Subsequent reversal of FlAsH binding is observed after 6 h with the addition of 5 mM EDT.

**References.**

1. Adams, S. R. & Tsien, R. Y. Preparation of the membrane-permeant biarsenicals FlAsH-EDT2 and ReAsH-EDT2 for fluorescent labeling of tetracysteine-tagged proteins. *Nat Protoc* **3**, 1527–1534 (2008).

2. Pokhrel, R., Tang, T. & Holub, J. M. Monitoring ligand-mediated helix 12 transitions within the human estrogen receptor α using bipartite tetracysteine display. *Org Biomol Chem* **18**, 6063–6071 (2020).

3. Gasteiger, E. *et al.* ExPASy: The proteomics server for in-depth protein knowledge and analysis. *Nucleic Acids Res* **31**, 3784–3788 (2003).

4. Eiler, S., Gangloff, M., Duclaud, S., Moras, D. & Ruff, M. Overexpression, purification, and crystal structure of native ER alpha LBD. *Protein Expr Purif* **22**, 165–173 (2001).

5. Bradford, M. M. A rapid and sensitive method for the quantitation of microgram quantities of protein utilizing the principle of protein-dye binding. *Anal Biochem* **72**, 248–254 (1976).

6. Adams, S. R. *et al.* New biarsenical ligands and tetracysteine motifs for protein labeling in vitro and in vivo: synthesis and biological applications. *J Am Chem Soc* **124**, 6063–6076 (2002).

7. Schneider, C. A., Rasband, W. S. & Eliceiri, K. W. NIH Image to ImageJ: 25 years of image analysis. *Nat Methods* **9**, 671–5 (2012).
